# The egg and larval pheromone dodecanoic acid mediates density-dependent oviposition of *Phlebotomus papatasi*

**DOI:** 10.1186/s13071-020-04151-w

**Published:** 2020-06-03

**Authors:** Dannielle Kowacich, Eduardo Hatano, Coby Schal, Loganathan Ponnusamy, Charles S. Apperson, Tatsiana Shymanovich, Gideon Wasserberg

**Affiliations:** 1 Department of Biology, University of North Carolina at Greensboro, 235 Eberhart Bldg., Greensboro, NC 27402 USA; 2 Department of Entomology and Plant Pathology, North Carolina State University, Raleigh, NC 27695 USA

**Keywords:** Leishmaniasis, Oviposition behavior, Sand flies, Semiochemicals, Dose-response bioassay, Oviposition regulation, Allee effect, Isovaleric acid

## Abstract

**Background:**

Gravid females assess the conditions of oviposition sites to secure the growth and survival of their offspring. Conspecific-occupied sites may signal suitable oviposition sites but may also impose risk due to competition or cannibalism at high population density or heterogeneous larval stage structure, respectively. Chemicals in the habitat, including chemicals emitted from other organisms, serve as cues for females to assess habitat conditions. Here, we investigated the attraction and oviposition preference of the Old World cutaneous leishmaniasis vector, *Phlebotomus papatasi*, to young and old conspecific stages, including eggs and evaluated the effect of a semiochemical associated with eggs and neonate larvae.

**Methods:**

Attraction and oviposition preference of *Ph. papatasi* to each of various life stages (eggs, first-, second-, third-, fourth-instar larvae, pupae and male and female adults) was investigated using cage and oviposition jar behavioral assays. Identification of organic chemical compounds extracted from eggs was performed using GC-MS and chemicals were tested in the same behavioral assays in a dose-response manner. Behavioral responses were statistically analyzed using logistic models.

**Results:**

Gravid *Ph. papatasi* females were significantly attracted to and preferred to oviposit on medium containing young life stages (eggs and first instars). This preference decreased towards older life stages. Dose effect of eggs indicated a hump-shaped response with respect to attraction but a concave-up pattern with respect to oviposition. Chemical analysis of semiochemicals from eggs and first-instar larvae revealed the presence of dodecanoic acid (DA) and isovaleric acid. Sand flies were attracted to and laid more eggs at the lowest DA dose tested followed by a negative dose-response.

**Conclusions:**

Findings corroborated our hypothesis that gravid sand flies should prefer early colonized oviposition sites as indicators of site suitability but avoid sites containing older stages as indicators of potential competition. Findings also supported the predictions of our hump-shaped oviposition regulation (HSR) model, with attraction to conspecific eggs at low-medium densities and switching to repellence at high egg densities. This oviposition behavior is mediated by DA that was identified from surface extracts of both eggs and first-instar larvae. Isovaleric acid was also found in extracts of both stages.
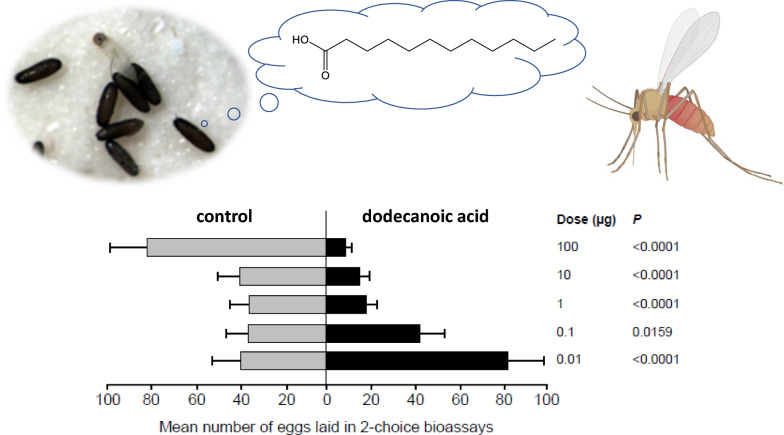

## Background

For organisms lacking parental care and with limited larval dispersal, oviposition site selection decisions are critical life-history choices [1–4]. For this type of organisms, the “Preference Performance Hypothesis” (PPH) posits that natural selection should favor oviposition behavior that optimizes offspring performance [5]. Most studies on factors affecting oviposition site selection in blood-feeding insects were done with mosquitoes and showed that, generally, gravid females avoid sites with predators/pathogens and are attracted to resource-rich sites [1, 2]. The effects of conspecific immature stages on oviposition site-selection of gravid mosquitoes are equivocal, with some studies reporting no effect, some reporting positive effects, some reporting negative effects and some reporting mixed-effects [1, 6–9]. To resolve this ambiguity, Wasserberg et al. [9] suggested a model that posits a trade-off between attracting/arresting cues at low densities that convey information about habitat suitability and repelling/deterring cues at high densities indicative of potential deleterious intraspecific competition. This model predicts a hump-shaped functional response between oviposition response and conspecific density, with preference for the conspecific-containing site initially increasing with density, peaking at some intermediate density and then decreasing and even becoming negative at higher densities. This hump-shaped density-dependent regulation model (HSR model), which is a form of the more general “Allee effect” [10], was supported by a comprehensive meta-analysis as well as field studies on *Aedes albopictus* indicating hump-shaped regulation at both the conspecific egg and larval stages. These relationships are mediated by a broad range of semiochemical cues [1, 2].

Different life stages are expected to differentially influence oviposition site selection of conspecific females. For example, motile larvae or adult ladybirds that might cannibalize the eggs were shown to have an inhibitory effect on oviposition, whereas non-motile eggs and pupae that pose no immediate risk to eggs do not affect female’s oviposition response [11]. Notably, Mwingira et al. [12] recently discovered that first-instar conspecific larvae induced a positive effect on oviposition response of *Anopheles coluzzii* while fourth-instar larvae induced a negative oviposition response, with deterrence from fourth-instar larvae explained in terms of cannibalism avoidance [12]. Interestingly, the density effect was unidirectional in both cases, with increasing density of first instars enhancing oviposition while increasing density of fourth instars enhancing deterrence.

Most research on oviposition attractants of disease vectors has focused on mosquitoes [1, 2, 9, 13] but relatively little is known about this topic with sand flies, vectors of human leishmaniasis worldwide [14–19]. For New and Old World sand flies, there is strong evidence that organic matter of various sources elicits oviposition responses, which makes adaptive sense given the coprophagic diet of the larvae [14, 16]. For example, for *Lutzomyia longipalpis*, a New World species, volatiles from rabbit and chicken feces were attractive to gravid females [20–23] and hexanal and 2-methyl-2-butanol were identified as active compounds [21]. Similarly, *Phlebotomus papatasi* (vector of cutaneous leishmaniasis in the Middle East) [22, 24–26] and *Phlebotomus argentipes* (vector of visceral leishmaniasis in India) [27] were shown to be strongly attracted and/or stimulated to oviposit by cow and rabbit feces and larval frass.

While the effect of larval food on guiding oviposition site choice is clear, the effect of conspecifics is less consistent. Most of the work in this field was done with *Lu. longipalpis*. Elnaiem & Ward [28] reported a positive effect of conspecific eggs on the oviposition response of *Lu. longipalpis*. This effect was dose-dependent but required a threshold of 160 eggs to exhibit this positive effect. An oviposition pheromone, initially recognized from the eggs, was isolated from female accessory glands [29] and identified as dodecanoic acid (DA) [30]. With Old World sand flies, Srinivasan et al. [31] reported positive effects of conspecific eggs on oviposition of *Ph. papatasi* and Wasserberg & Rowton [22] confirmed a positive but weak effect of conspecific eggs on oviposition response. Similarly, conspecific eggs were reported to have a positive effect on oviposition response of *Ph. argentipes* [32]. Notably, because all these studies used oviposition bioassays, it remains unclear whether the oviposition responses are guided by contact chemo-stimuli, volatile attractants, or both.

The effect of conspecific stages on the oviposition-site selection of sand flies has focused predominantly, as discussed above, on conspecific eggs. Very little attention was given to other conspecific stages. Basimike [33] studied the oviposition responses of three species of Old World sand flies (*Sergentomyia ingrami*, *Sergentomyia schwetzi* and *Phlebotomus duboscqi*) to crushed eggs and smeared samples of larvae (first- and fourth-instar), pupae and adult males and females. He also evaluated the oviposition responses of *S. ingrami* to immature and mature stages of *Ph. duboscqi*. With *S. ingrami*, Basimike [33] observed a strong positive effect of eggs, fourth-instar larvae, pupae and adult males and females but not for first-instar larvae. With *S. schwetzi*, he observed a strong positive effect of all stages and with *Ph. duboscqi* he also observed a strong effect of all stages except for fourth-instars. When looking at cross-species effects, Basimike [33] reported a significant positive effect of adult females and a generally positive but weak and non-significant effect of all other stages. In contrast, Schlein et al. [34] reported that larvae (third-/fourth-instar) and pupae had a strong negative effect on the oviposition responses of *Ph. papatasi.* Thus, no clear pattern emerges with respect to the potential adaptive value of differential responses to different conspecific stages. Because sand flies are characterized by a fully terrestrial life-cycle with coprophagic larvae typically relying on decomposing organic matter as food source [14, 16], it is plausible that gravid females would be attracted to early juvenile stages, such as eggs or first instars, as indicative of a suitable but relatively underused oviposition site. In contrast, gravid females might be repelled from older conspecific stages such as fourth-instar larvae, pupae, or adults as indicators of a low-quality, resource-depleted oviposition site. An alternative, but not mutually exclusive, hypothesis is that, as in the case of ladybirds [11], gravid females might not respond to non-motile conspecific stages such as eggs or pupae that pose no cannibalistic threat to their neonates, but they might avoid larger and older stages that are known to cannibalize eggs and young larvae [35].

In this study, we separated oviposition responses into their two main stages, attraction to oviposition-site cues and stimulation of oviposition. In oviposition experiments, sand flies contacted an oviposition substrate and were exposed to volatile and contact pheromones emitted by various life stages. In attraction experiments, the same life stages, extracts, or compounds were assayed in a free flight arena using sticky traps where sand flies could respond only to volatile compounds emitted from the test materials. We first evaluated the oviposition and attraction responses of gravid *Ph. papatasi* sand flies to conspecific immature and adult stages. Then, after identifying conspecific eggs as a source for both oviposition stimulation and attraction, we conducted dose-response studies with both assays. Based on the HSR model [9], we hypothesized that oviposition and attraction would be positively affected at low-to-intermediate doses but negatively affected at high doses. Volatile chemical profile analysis identified DA as the main component in egg surface extracts and dose-response oviposition and attraction bioassays followed.

## Methods

### Insects and colony maintenance

*Phlebotomus papatasi* sand flies that originated from Abkük, Turkey (April, 2004) were maintained following the mass-rearing technique described by Lawyer et al. [36]. The adults were blood-fed at SoBran (Greensboro, NC, USA) on live anesthetized ICR mice (Envigo, Indianapolis, IN, USA) (SoBran protocol # UNC-002-2016). Sand flies were maintained in incubators (Caron^®^, Marietta, OH, USA) at 27 °C, 85% RH under a 14:10 h light:dark reverse photoperiod, with 1 h of crepuscular light conditions (using an 11 W incandescent light bulb connected to an automatic timer) at the start of the light phase representing twilight at sunrise (dawn) and 1 h at the end of the light phase representing twilight at sunset (dusk) [37].

### Conspecific stages effect: attraction bioassays

**Fig. 1:**
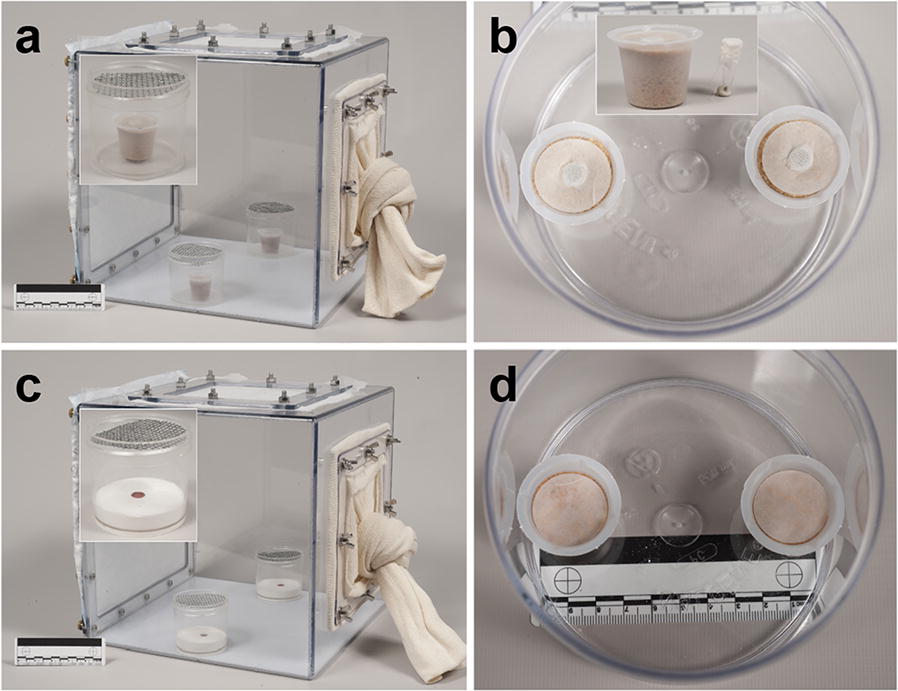
Design of bioassay chambers and oviposition attractants used to assay responses of gravid *Ph. papatasi*. **a** Attraction bioassay using sticky traps for testing various conspecific stages, including eggs. The inset shows a magnified image of the sticky trap showing a stimulus-containing micro-centrifuge tube embedded in the sand in the sand cup. A sticky metal screen was positioned above the treatment and control cups. **b** Oviposition bioassay for testing the effect of conspecific stages, including eggs, on number of eggs deposited by gravid females. The top view shows the screened top of the microcentrifuge tube that contains the stimulus and filter paper that serves as oviposition substrate. **c** Attraction bioassay chamber using sticky traps for testing attraction to dodecanoic acid. The inset shows the rubber septum embedded in plaster. Dodecanoic acid was applied directly to the septum to allow for slow release. The sticky metal screen positioned above the treatment and control cup traps females attracted to the septa. **d** Oviposition bioassay for testing the effect of dodecanoic acid. Dodecanoic acid was applied directly on the filter paper and acetone was applied to the control filter paper. Females oviposited on the filter papers. Image credit: Daniel J. Smith

To evaluate the attraction of gravid *Ph. papatasi* females to conspecific material, we conducted a two-choice bioassay using 30 × 30 × 30 cm polycarbonate free-flight cages consisting of a pair of sticky traps (Fig. 1a). These are 125 ml Nalgene jars (Nalgene™, Rochester, NY, USA; Model 1187580, diameter = 6.5 cm); one containing the treatment sand cup (described below) and the other contained the control sand cup. A metal screen, previously sprayed with an adhesive (Tanglefoot^®^, Grand Rapids, MI, USA; Model 91992-MI-001) was then placed on top of each jar (Fig. 1a). Sand cups are 10 ml disposable beakers (Thermo Fisher Scientific^®^, Waltham, MA, USA; Model: 08-732-121) filled with 8 ml of autoclaved sand and 2.5 ml of deionized (DI) water to keep the sand moist (Fig. 1a). A stimulus-containing microcentrifuge tube (Axygen, Inc., Corning, NY, USA; Model 321-02-051) was pushed through the sand to be flush with the level of the sand (Fig. 1b, top). Treatment tubes contained 0.6 mg of each stimulus type and covered with a double nylon fabric mesh and a clear rubber band to preclude visual cues. Control cups consisted of a similar but empty tube. The following live conspecific stages were obtained from our sand fly rearing colony: fresh eggs, first-instar larvae, second-/third-instar larvae, fourth-instar larvae, pupae, adult males and gravid females. These were thoroughly cleaned by brushing off rearing material and debris. Twenty gravid female sand flies, approximately 5.5 days post-blood meal, were then transferred into the cage 24 h prior to the experiment to get acclimated to assay conditions. The experiment was conducted in a dark (illuminated with two red incandescent bulbs) climate control room set at 27 °C, 65% RH. After 24 h, the number of flies attached to each adhesive metal screen was counted. Six replicate sessions where conducted, with 2–4 replicates for each stage per session.

### Conspecific stages effect: oviposition bioassays

To determine the effects of conspecific material on total oviposition response of *Ph. papatasi* a two-choice oviposition bioassay was used, with a pair of sand cups placed inside a 500 ml Nalgene jar (Nalgene™, diameter = 11 cm) (Fig. 1b). Sand cups were similar to those described above only here we also used a wet filter paper as an oviposition substrate. Specifically, 2.5 cm in diameter filter paper (Thermo Fisher Scientific^®^) with a centered 0.5 cm hole was placed on top of the sand cups and wetted with 0.25 ml of DI water to saturate the filter paper. A stimulus-containing microcentrifuge tube was then pushed through the hole to be flush with the level of the filter paper (Fig. 1b). As above, treatment tubes contained 0.6 mg of each stimulus type and covered with a double nylon fabric mesh and a clear rubber band to prevent visual cues (Fig. 1b, top). Control cups consisted of a similar but empty tube. The same conspecific stages were prepared and used: fresh conspecific eggs, first-instar larvae, second-/third-instar larvae, fourth-instar larvae, pupae, adult males and gravid females. One treatment and one control sand cups were placed in opposite sides of the 500 ml Nalgene jar and covered with nylon fabric mesh secured with a pair of rubber bands (Fig. 1b). Twenty females, approximately 7 days post-blood meal, were then inserted into the jar with a mouth aspirator. Jars were then placed in a randomly assigned location in a 55 × 40 cm plastic tub. The experiment ran for three days in the colony incubators under standard larval rearing conditions. Every 24 h, 0.25 ml of DI water was added to each sand cup to keep the sand and filter paper moist and fresh sugar pads (cotton ball soaked in 30% sucrose solution) were provided. After the 3-day period, flies were removed and the eggs laid on the filter papers were counted under a dissection microscope (Olympus SZ61, Center Valley, PA, USA, 6.7×–45×). Six replicate sessions where conducted, with three replicates for each conspecific stage per session (total *n* =18 for each conspecific stage).

### Egg dose effect

After determining that eggs elicited a consistent strong positive effect for both oviposition and attraction, we conducted a dose-response series of the effect of egg mass on oviposition response and attraction. A range of 5 different egg masses were used (0.15 mg, 0.3 mg, 0.6 mg, 1.2 mg, 2.4 mg) placed inside microcentrifuge tubes (Fig. 1b). Eggs were weighed on a Mettler Toledo©, XSE105 scale (Columbus, OH, USA). The oviposition bioassay was done as described above, but the attraction bioassay was shortened to 6 h from 12:00 to 18:00 h representing the second half of the scotophase shown recently to be the optimal time for studying oviposition attraction [37]. For both oviposition and attraction bioassays, four replicate sessions where conducted with 5 replicates for each egg mass category (total *n* = 20 for each egg mass category).

### Chemical analysis of *Ph. papatasi* eggs and first-instar larvae

*Phlebotomus papatasi* eggs were collected from colony jars by immersing the rearing material in distilled water to suspend eggs. Eggs were then collected by pouring the water into a sieve (Hogentogler, Columbia, MD, USA, 90 μm mesh, No. 170) and rinsed with distilled water before eggs were placed on a filter paper (Fisherbrand, Toronto, ON, Canada, 18.5 cm, No. 9-803-5G) to dry for 30 min. A mass of 4.15 mg of eggs (approximately 1400 eggs) was transferred to a clear borosilicate vial (12 × 32 mm, National, Rockwood, TN, USA) and left for 12 h at 27 °C to reconstitute compounds that may have been washed during the egg collection. First-instar larvae were collected from a separate colony jar under a stereomicroscope (Olympus SZ61, 6.7×) using a fine brush and gently cleaned of debris over a filter paper. A mass of 1.5 mg of larvae (approximately 88 larvae) was transferred to a clear borosilicate vial and extracted as follows. Acetone (200 μl, Sigma-Aldrich, St. Louis, MO, USA) containing 2 μg tridecanoic acid as internal standard (IS) was added to vials containing either eggs or larvae. Preliminary analysis showed that tridecanoic acid was not present in egg nor in first-instar extracts. Vials were gently swirled for 1 min and left to rest for 5 min. Each extract was transferred to a second vial using a Pasteur pipette. This extraction procedure was repeated twice more using acetone without IS and the three extracts of each material were combined in the same vial. The egg and larval extracts were concentrated under a gentle flow of nitrogen (Airgas National Welders, Radnor, PA, USA) down to approximately 40 and 10 μl, respectively. An aliquot of 1 μl of each extract was injected into a gas chromatograph coupled to a mass spectrometer (GC-MS) for analysis. Control extracts were generated by following the same extraction protocol with clean vials but without eggs or larvae.

The GC-MS (6890 GC and 5975 MS, Agilent Technologies, Palo Alto, CA, USA) was equipped with a DB-WAXetr column (30 m × 0.25 mm, df = 0.25 μm, Agilent Technologies) and helium was used as the carrier gas at an average velocity of 34 cm/s. Oven program was set to 40 °C for 2 min, increased at 10 °C/min to 265 °C and held for 13 min. The injector was set to splitless mode (10 psi) at 265 °C, transfer line was also at 265 °C, MS source was set to 230 °C and the quadrupole was set to 150 °C. The mass-to-charge ratio range was 33 to 650. Compounds were identified based on Kovats indices, electron ionization mass spectra, and comparison and co-injection with authentic synthetic standards.

### Dodecanoic acid dose-response bioassays

After detecting DA in eggs and first-instar larvae, we investigated whether it had a dose-dependent effect on oviposition response and attraction. Dodecanoic acid (Supelco, Bellefonte, PA, USA) was diluted in hexane (Sigma-Aldrich) at concentrations of 0.001, 0.01, 0.1, 1 and 10 µg/µl and kept in a − 18 °C freezer until used. For all dose-dependent bioassays, five DA doses were used: 0.01 µg, 0.1 µg, 1 µg, 10 µg and 100 µg. For attraction bioassays, we used small Nalgene jars with a plaster-of-Paris bottom (1 cm high). An approximately 1 cm deep and 0.5 cm diameter hole was drilled into the plaster and a rubber septum (Wheaton, Millville, NJ) was inserted such that its surface was flush with the plaster surface (Fig. 1c). These septa were used to enable slow release of DA volatiles. Dodecanoic acid was applied to the septa, the sticky mesh was replaced on top of the jar, and jars inserted to free flight chamber. The experiment was conducted in a climate control room at 27 °C, 65% RH, for a 6-h period. After 24 h the number of flies attached to each adhesive metal screen was counted. For attraction and oviposition bioassays, two replicate sessions where conducted with 6 and 8 replicates per DA dose category, respectively (total *n* =12 and *n* = 16 for each DA category, respectively). Oviposition bioassay settings were, basically, similar to those described above only that here DA was applied directly to dry filter paper and an equivalent amount of hexane (10 µl) applied to the control filter paper (Fig. 1d). Filter papers were left for 5 min for the solvent to evaporate. The experiment ran for three days in the colony incubators under standard rearing conditions; every 24 h fresh sugar pads were provided. After the 3-day period, flies were removed and the eggs laid on the control and treated sand cups were counted under a dissection microscope (Olympus SZ61, 6.7×–45×).

### Data reduction and statistical analysis

In order to assess the affinity of sand flies to our candidate materials or DA relative to their affinity to the control, we calculated an oviposition preference index, which is the number of flies caught (or eggs laid) in the treatment jar divided by total flies trapped (or eggs laid) in control and treatment jars combined. Values above 0.5 indicated affinity for the treatment and values below 0.5 indicated aversion to it. Given that “preference” is a proportion, we analyzed these data using weighted logistic regression, with the total number of flies trapped in the treatment and control sticky traps combined or the total number of eggs laid in the treatment and control oviposition jars combined, as the weighting factor. Based on this logistic regression analysis, we estimated the mean value of the odds (± 95% CI) for each material tested and its significance level. To test for linear or non-linear relations between preference and conspecific age-class and for egg or DA amount, we tested three candidate models: (i) a simple linear model; (ii) a second-order polynomial model with a non-zero intercept; and (iii) a second-order polynomial model with a zero-intercept and selected the best model based on its AIC (Akaike Information Criterion) score. Specifically, we coded egg-to-adult stages as 0-to-5 and ran the regression once with adult males and females combined and then separately with either adult males or gravid females as stage 5. Egg mass and DA dose were used as a continuous variable but were log-transformed (with a + 1 and + 3 offset, respectively, to avoid negative values) to control for potential statistical leverage issues.

## Results

### Effects of conspecific stages on attraction and oviposition responses

#### Attraction

**Fig. 2:**
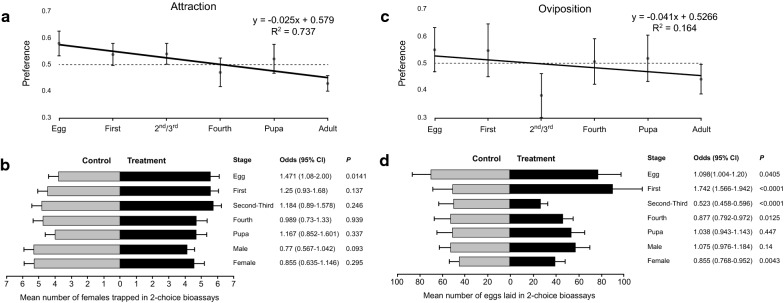
Attraction (**a**, **b**) and oviposition (**c**, **d**) responses of *Ph. papatasi* to conspecific stages. Top panels (**a**, **c**) depict the least square regression lines of oviposition site preferences (± standard error, SE) based on attraction (**a**) or oviposition (**c**), against ordinal conspecific stages arranged from youngest (eggs) to the oldest stage (adults). In this analysis adult males and females were combined. Horizontal dashed line crossing through the preference value of 0.5 indicates neutral preference with values above or below this line indicating positive or negative effects of conspecific stage, respectively. Bottom panels (**b**, **d**) depict the mean abundance (± SE) of females trapped (**b**) or eggs laid (**d**) in control and treatment cups. Also depicted are the odds (with their respective 95% CI and *P*-value) of females trapped (**b**) or eggs laid (**d**) in the treatment cup compared with the control cup

A clear and significant negative linear relationship was observed between conspecific stage (used as an ordinal variable) and oviposition site preference (Table 1), with attraction observed towards the younger stages and then gradually decreasing toward (non-significant) repellence from older stages (Fig. 2a, b). This negative linear effect was also significant when using either adult males (*P* = 0.005) or gravid females (*P* = 0.014) as the oldest stage. Specifically, eggs were the only stage significantly attractive to gravid females, followed by non-significant attraction to first- and second-/third-instar larvae (Fig. 2b). Fourth instars and larvae had a neutral effect, while adults had a non-significant (females) or a marginally significant (males) repellent effect (Fig. 2b). Experimental data are provided in Additional file 1: Table S1.

**Table 1:** The effect of conspecific stages on the attraction and oviposition response of gravid sand flies: weighted logistic regression table

Bioassay	Variable	Coefficient (SE)	*Z-*value	*P*-value
Attraction	Stage	− 0.090 (0.03)	− 2.45	0.014
	Intercept	0.349 (0.11)	3.14	0.001
Oviposition	Stage	− 0.041 (0.01)	− 3.975	< 0.0001
	Intercept	0.127 (0.03)	3.638	0.0002

*Note*: Conspecific stages were coded as ordinal variables: eggs (0), first-instars (1), second-/third-instars (2), fourth-instars (3), pupae (4) and adults (5)

#### Oviposition

A significant negative linear relationship was observed between conspecific stage and oviposition response (Table 1), with positive oviposition response observed towards the younger stages and then gradually decreasing toward deterrence from older stages (Fig. 2c, d). This effect was also significant when using either adult males (*P* = 0.004) or females (*P* < 0.0001) as the oldest stage. Specifically, significant oviposition stimulation was induced by eggs and especially by first-instar larvae (Fig. 2d). In contrast, a strong and significant deterrent effect was observed with respect to second-/third-instar larvae and to a lesser extent, by fourth-instar larvae. Pupae and adult males had a neutral effect, but adult females had a significant deterring effect on oviposition (Fig. 2d). Experimental data are provided in Additional file 2: Table S2.

### Dose-response effects of conspecific eggs on attraction and oviposition responses

#### Attraction

**Fig. 3:**
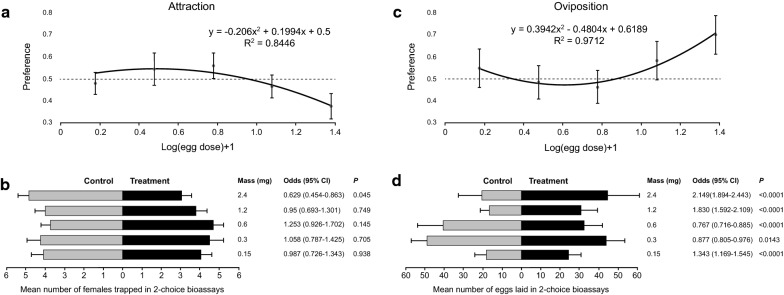
Attraction (**a**, **b**) and oviposition (**c**, **d**) responses of *Ph. papatasi* to different doses of conspecific eggs. Top panels (**a**, **c**) depict the least square regression lines of oviposition site preferences (± SE) of the best-fit models, based on attraction (**a**) or oviposition (**c**), against conspecific egg mass (mg) ((log x)+1 transformed). Horizontal dashed line crossing through the preference value of 0.5 indicates no preference with values above or below this line indicating positive or negative effects of conspecific eggs, respectively. Bottom panels (**b**, **d**) depict mean (± SE) abundance of females trapped (**b**) or eggs laid (**d**) in control and treatment oviposition cups, respectively. Also shown are the odds (with their respective 95% CI and *P*-value) of females trapped or eggs laid in the treatment cup compared with the control cup

A clear hump-shaped relationship between egg dose and oviposition site preference was observed (Fig. 3a). The best-fit model was a second-order polynomial model with zero intercept (Table 2). This model was slightly better than a similar non-zero intercept model (ΔAIC = 0.87) but quite better than a linear model (ΔAIC = 4.6). Based on this model, preference is estimated to peak at 0.32 mg of eggs (approximately 119 eggs) and to switch from attraction to repellence at 0.92 mg of eggs (approximately 342 eggs). None of the individual egg doses used had a significant attractive effect and the only significant effect was repellency at the highest dose (Fig. 3b). Experimental data are provided in Additional file 3: Table S3.

**Table 2:** The effect of egg dose on the attraction and oviposition responses of gravid sand flies: second-order polynomial weighted logistic regression table

Bioassay	Variable	Coefficient (SE)	*Z*-value	*P*-value
Attraction	Egg dose	0.682 (0.32)	2.13	0.032
	Egg dose^2^	− 0.710 (0.28)	− 2.57	0.010
Oviposition	Egg dose	− 2.236 (0.03)	− 7.38	< 0.0001
	Egg dose^2^	1.791 (0.18)	9.65	< 0.0001
	Intercept	0.566 (0.10)	5.32	< 0.0001

#### Oviposition

Concave-up relationship between egg dose and oviposition site preference was observed (Fig. 3c), with best-fitting logistic model being a second order polynomial model (Table 2). Specifically, at the lowest egg dose of 0.15 mg significant positive stimulatory effect of eggs was detected, which then switched to significant deterrence at 0.3 and 0.6 mg, and then switched back to significant oviposition stimulation at 1.2 mg with strongest stimulatory effect at 2.4 mg eggs (Fig. 3d). Experimental data are provided in Additional file 4: Table S4.

### Chemical analysis of *Ph. papatasi* eggs

**Fig. 4:**
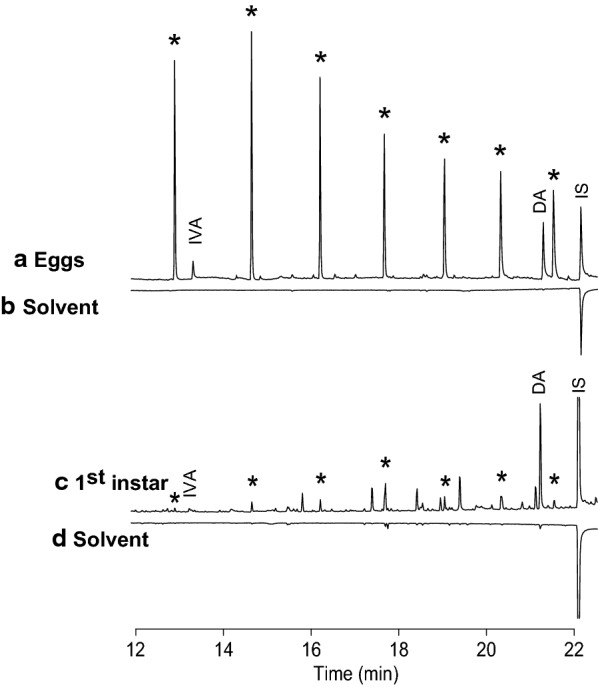
Gas chromatography-mass spectrometry analysis of extracts of *Ph. papatasi* eggs and first instars. Eggs (4.15 mg) were extracted with acetone containing 2 µg tridecanoic acid as internal standard (IS). Total ion chromatogram of egg extract (**a**) and extract of first-instars (**c**) are depicted. Mirrored downward chromatograms (**b**, **d**) show the respective acetone control. Dodecanoic acid (DA) and isovaleric acid (IVA) were identified. The retention times of the analyses in (**c**) and (**d**) are slightly different from those in (**a**) and (**b**) because they were run at different times. *Cyclosiloxane contaminants from plastic and plaster

Analysis of acetone extracts of *Ph. papatasi* eggs, identified dodecanoic acid (DA) and isovaleric acid at concentrations of 280 and 101 ng/mg eggs, respectively (0.76 and 0.27 ng/egg, respectively) (Fig. 4a, upward chromatogram). Dodecanoic acid and isovaleric acid were also detected in first-instar larvae extracts at concentrations of 32.6 and 1.39 ng/mg larvae, respectively (0.56 and 0.024 ng/larva, respectively) (Fig. 4c). These compounds were not present in acetone alone (Fig. 4b, d, downward chromatograms). In addition, analyses showed many cyclosiloxanes as contaminants from the plastic and plaster of our rearing containers. Since DA was the major compound in the extracts, and it was shown to stimulate oviposition in *Lu. longipalpis* [29], we tested the bioactivity of DA in oviposition and attraction bioassays with *Ph. papatasi* gravid females.

### Dose-response effects of dodecanoic acid on attraction and oviposition responses

#### Attraction

**Fig. 5:**
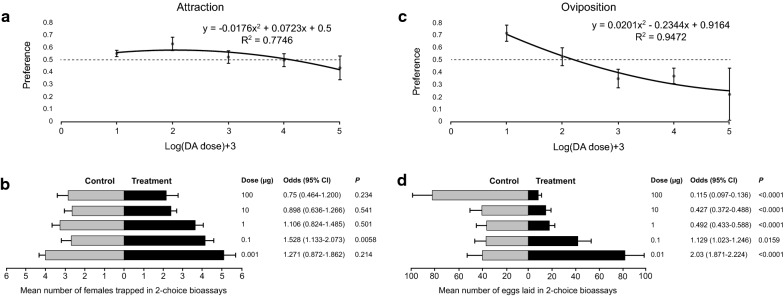
Attraction (**a**, **b**) and oviposition (**c**, **d**) responses of *Ph. papatasi* to different doses of dodecanoic acid. Top panels (**a**, **c**) depict the least square regression lines of oviposition site preferences (± SE) of the best-fit models, based on attraction (**a**) or oviposition (**c**), against dodecanoic acid dose (µg) (log (x)+3 transformed). Horizontal dashed line crossing through the preference value of 0.5 indicates no preference with values above or below this line indicating positive or negative effects of DA dose, respectively. Bottom panels (**b**, **d**) depict mean (± SE) abundance of females trapped (**b**) or eggs laid (**d**) in control and treatment oviposition cups, respectively. Also shown are the odds (with their respective 95% CI and *P*-value) of females trapped or eggs laid in the treatment cup compared with the control cup

A hump-shaped relationship between DA dose and oviposition site preference was observed (Fig. 5a). The best-fit model was that of a second-order polynomial model with zero intercept (Table 3). Yet, this model was only slightly better than a negatively sloped linear model (ΔAIC = 0.3) but quite better than a second-order polynomial model without a zero intercept (ΔAIC = 1.6). Based on the best-fit model, preference is estimated to peak at 0.13 µg DA and to switch from attraction to repellence at 13 µg DA (Fig. 5a). Non-significant attraction was observed at the lowest dose of 0.01 µg, followed by significant attraction at 0.1 µg DA. Attraction then decreased to non-significant attraction at 1 µg DA followed by non-significant repellence at 10 and 100 µg (Fig. 5b). Experimental data are provided in Additional file 5: Table S5.

**Table 3:** The effect of dodecanoic acid dose on the attraction and oviposition responses of gravid sand flies: second-order polynomial weighted logistic regression table

Bioassay	Variable	Coefficient (SE)	*Z*-value	*P*-value
Attraction	DA dose	0.296 (0.10)	3.10	0.002
	DA dose^2^	− 0.076 (0.02)	− 3.08	0.002
Oviposition	DA dose	− 0.461 (0.09)	− 4.99	< 0.0001
	DA dose^2^	− 0.034 (0.02)	− 2.15	0.032
	Intercept	1.190 (0.11)	10.95	< 0.0001

#### Oviposition

A clear negative relationship between oviposition site preference and DA dose was observed (Fig. 5c). This relationship was best described using a decelerating second-order polynomial model (Table 3) with ΔAIC > 2 with respect to other models. Based on this model, preference is estimated to switch from oviposition stimulation to deterrence at 0.15 µg DA (Fig. 5c). Specifically, *Ph. papatasi* laid significantly more eggs in cups with 0.01 µg and 0.1 µg DA than in the respective control cups (Fig. 5d). However, this preference dropped sharply to significant deterrence at the higher doses (Fig. 5d). Experimental data are provided in Additional file 6: Table S6.

## Discussion

In this study, we evaluated the attraction and oviposition responses of gravid *Ph. papatasi* sand flies to conspecific immature and adult stages. Next, after identifying that conspecific eggs attracted gravid females and stimulated oviposition, we evaluated the dose-response effect of conspecific eggs on sand fly attraction and oviposition. We identified dodecanoic acid as a putative egg and first-instar larvae pheromone that guides female oviposition choices, and conducted dose-response studies with it, validating its effect as a pheromone that attracts gravid females and stimulates oviposition.

### Differential effects of conspecific stages

Gravid females were attracted to and stimulated by early immature stages (eggs and first-instar larvae) but were not affected, or even deterred by later stages. These results suggest that younger immature stages may provide “reassurance” cues indicative of a suitable and favorable oviposition site that is relatively underexploited, while the presence of older conspecific stages may communicate that this oviposition site is resource-depleted and risky (due to cannibalism). The positive effect of conspecific eggs on oviposition response is consistent with previous studies [22, 28–33, 38], some of which also identified DA as the oviposition pheromone [30]. However, these studies did not distinguish attraction to volatile compounds from oviposition triggered by contact. Our study indicates a qualitatively similar negative effect of conspecific stage age class on both attraction and oviposition. Moreover, as far as we know, the positive stimulatory effect of first instars on oviposition has not been previously demonstrated for sand flies. Furthermore, our study is the first to demonstrate that, in addition to eggs, first-instar larvae also emit DA. Another compound isolated, for the first time, from both eggs and first-instar larvae is isovaleric acid, which is also produced by an attractive saprophytic bacterium that we recently isolated (unpublished data) from an attractive larval rearing medium [24]. We have shown that conspecific eggs produce these two compounds but it is not yet clear if first-instar larvae are producing these compounds or acquire them by feeding on egg chorions [39] or possibly by being “anointed” with egg-specific pheromone. The presence of isovaleric acid in eggs (pheromone) and rearing medium (kairomone) may suggest evolutionary parsimony with isovaleric acid indicating a suitable oviposition site in both contexts.

The strong deterrent effect of older larvae and particularly of second-/third-instars, on oviposition responses may be a female strategy of avoiding cannibalism of her neonate offspring by older, larger and more feeding-active larval stages [35]. Our results are consistent with Schlein et al. [25] who worked with the same species (*Ph. papatasi*) and reported oviposition deterrence from third- and fourth-instar larvae. Interestingly, in three other African sand fly species, all conspecific stages, including older larval stages, stimulated oviposition [33]. However, these results might be related to the use of crushed samples possibly releasing their excreta and gut microbes that might be attractive and/or stimulate oviposition [24].

The strong oviposition deterrent effect of second-/third-instar larvae observed here is particularly interesting because in a previous study [24], we showed that rearing medium of second-/third-instar larvae was the most attractive and oviposition stimulating medium compared with virgin rearing media or rearing media of older stages (note that in those experiments rearing media tested did not contain any larvae). That attraction was shown to be driven by bacterial odorants (unpublished data). When put together, these findings suggest that gravid females encountering a potential oviposition site (an organic matter-rich patch) might be faced with two opposing stimuli: a deterrent stimulus from older larval stages, communicating potential risk of cannibalism, and a positive stimulatory effect from saprophytic or gut bacteria, indicating availability of food for their progeny. The nature of the interplay between these two opposing forces warrants further investigation.

### The dose-dependent effect of conspecific eggs

The HSR model posits a trade-off between habitat suitability reassurance cues of conspecifics at low densities but repelling/deterring cues at high densities indicative of potential deleterious intraspecific effects [9]. Results of the attraction bioassays were consistent with this hypothesis, exhibiting a hump-shaped relationship between egg mass and preference for the eggs-containing-jar with neutral preference at low (0.15 mg) egg density, increasing and peaking at 0.6 mg, and then declining sharply to repellency at the highest egg densities (1.2 and 2.4 mg). This is the first report of hump-shaped oviposition regulation in sand flies. In contrast, results of the oviposition bioassays show an almost opposite concave-up pattern. The differential response of oviposition and attraction with respect to conspecific egg density suggests that these behaviors are driven by different compounds with opposite effects. This issue warrants further investigation.

### An egg and first-instar pheromone guides sand fly oviposition behavior

We identified dodecanoic acid as the most prevalent organic compound in acetone surface extracts of eggs and first-instar larvae. This result is consistent with previous findings of other researchers who worked on *Lu. longipalpis* [29, 30, 38], although they did not work with first-instar larvae. They extracted this pheromone from conspecific eggs and from females’ accessory glands and demonstrated a significant oviposition stimulatory effect of this pheromone [30]. Here, we observed a clear negative dose-dependent effect of dodecanoic acid on both attraction and oviposition with strong attraction and oviposition stimulation at low doses (0.01–0.1 µg) then switching to neutrality and repellence/deterrence at the higher doses. This switch was particularly apparent for oviposition. With respect to attraction, the DA hump-shaped dose-response curve was similar to that of the egg density effect, suggesting that the latter might be driven by DA. This was not the case with respect to oviposition, with egg density and DA dose having positive and negative effects on number of eggs laid, respectively. These results suggest that the positive effect of egg density may be mediated by a different semiochemical. Another interesting observation is that the effects of DA on attraction and oviposition were qualitatively different. Dodecanoic acid had a hump-shaped effect in attraction assays, and an exponential decay effect in oviposition assays. This suggests that, as discussed above, attraction and oviposition may be driven by different, but potentially overlapping, blends of semiochemicals.

## Conclusions

Our findings support our hypothesis that gravid sand flies should prefer early colonized oviposition substrates as indicators of site suitability but avoid substrates containing older stages as indicators of potential competition. Our findings are also consistent with the predictions of our HSR model, with attraction to conspecific eggs at low-medium densities of eggs and avoidance of high egg densities. The affinity to both conspecific eggs and first-instar larvae is mediated by dodecanoic acid. We also found isovaleric acid in extracts of eggs and first-instar larvae, but its function remains unknown. Further studies will focus on the combined effects of dodecanoic and isovaleric acids and other semiochemicals in attracting gravid females and stimulating oviposition.

## Supplementary information

**Additional file 1: Table S1.** Attraction of sand flies to conspecific stages.

**Additional file 2 :Table S2.** Oviposition of sand flies to conspecific stages.

**Additional file 3: Table S3.** Attraction to doses of conspecific eggs.

**Additional file 4: Table S4.** Oviposition response to doses of conspecific eggs.

**Additional file 5: Table S5.** Attraction to different doses of dodecanoic acid.

**Additional file 6: Table S6.** Oviposition response to doses of dodecanoic acid.

## Data Availability

All data generated or analyzed during this study are included in this published article and its additional files.

## Abbreviations

DA: dodecanoic acid
HSR: hump-shaped oviposition regulation
